# Intravital Fluorescence Excitation in Whole-Animal Optical Imaging

**DOI:** 10.1371/journal.pone.0149932

**Published:** 2016-02-22

**Authors:** Fatemeh Nooshabadi, Hee-Jeong Yang, Joel N. Bixler, Ying Kong, Jeffrey D. Cirillo, Kristen C. Maitland

**Affiliations:** 1 Department of Biomedical Engineering, Texas A&M University, College Station, Texas, United States of America; 2 Department of Microbial Pathogenesis and Immunology, Texas A&M University Health Science Center, Bryan, Texas, United States of America; Glasgow University, UNITED KINGDOM

## Abstract

Whole-animal fluorescence imaging with recombinant or fluorescently-tagged pathogens or cells enables real-time analysis of disease progression and treatment response in live animals. Tissue absorption limits penetration of fluorescence excitation light, particularly in the visible wavelength range, resulting in reduced sensitivity to deep targets. Here, we demonstrate the use of an optical fiber bundle to deliver light into the mouse lung to excite fluorescent bacteria, circumventing tissue absorption of excitation light in whole-animal imaging. We present the use of this technology to improve detection of recombinant reporter strains of tdTomato-expressing *Mycobacterium bovis* BCG (Bacillus Calmette Guerin) bacteria in the mouse lung. A microendoscope was integrated into a whole-animal fluorescence imager to enable intravital excitation in the mouse lung with whole-animal detection. Using this technique, the threshold of detection was measured as 10^3^ colony forming units (CFU) during pulmonary infection. In comparison, the threshold of detection for whole-animal fluorescence imaging using standard epi-illumination was greater than 10^6^ CFU.

## Introduction

Whole-animal optical imaging has found widespread use in the biological sciences. Fluorescence-based techniques have long been used to study both fixed and live biological specimens, as it allows for highly selective and specific detection of molecules even at low concentrations [1]. While traditional fluorescence microscopy requires the sacrifice of multiple animals at discrete time points, whole-animal imaging allows researchers to study disease progression or the efficacy of a therapeutic agent more precisely and with fewer animals than prior imaging techniques [2]. Whole-animal fluorescence imaging is used to study disease pathogenesis, develop novel probes, and measure response to new therapies [3]. Fluorescent reporter strategies include delivery of fluorescent probes targeted to a specific moiety or transfection of cells with genes encoded for a fluorescent protein as an intrinsic reporter probe.

To record a planar image of an internal fluorescent target in a small animal, whole-animal imaging systems can be configured either for epi-illumination, with the light source on the same side of the tissue as the detector, or trans-illumination, with source and detector on opposite sides of the tissue. Although trans-illumination is preferable due to superior tissue volume sampling and reduced skin autofluorescence background, trans-illumination systems are more complex and expensive than epi-illumination. Fluorescence molecular tomography using multiple source and/or detector locations provides further enhancements in sensitivity and localization of fluorescent targets over planar trans-illumination [4].

Simplicity in design, ease of operation, and high throughput has yielded broad use of epi-illumination imaging systems, albeit with noteworthy shortcomings. Excitation light is generated from a filtered lamp and broadly distributed over the sample stage to provide relatively even field illumination. Surface and subsurface fluorescence signal emitted from the animal is filtered by high-quality emission filters and captured by a sensitive charge-coupled device (CCD) camera. Epi-illumination systems traditionally suffer from relatively poor depth sensitivity due to light absorption by tissue [3]. Excitation light decreases exponentially with depth; thus, the images are surface-weighted. This is particularly the case when using fluorescent reporters that excite with shorter wavelengths (<600 nm) where hemoglobin absorption is stronger [5]. Additionally, autofluorescence from a wide variety of molecules present in living tissue, particularly the skin in epi-illumination mode, generates significant background signal in this portion of the spectrum [6]. Increased background limits detection sensitivity, especially when the fluorescent target is deep within the animal [7]. Therefore, detected fluorescence signal depends on the brightness and excitation wavelength of the selected fluorescent protein, the number of fluorescent cells, and the depth and location within the animal.

Whole-animal imaging has been used to study a wide range of diseases and organ systems, including brain cancer [8], neurological degenerative disease [9], atherosclerosis [10], myocardial infarction [10, 11], and respiratory infections such as tuberculosis [12]. For many applications, increased detection sensitivity could greatly enhance the ability to study pathogenesis in more physiologically relevant conditions. For instance, detection of *Mycobacterium tuberculosis* (Mtb) expressing tdTomato fluorescent protein has been demonstrated *in vivo* for subcutaneous infections of 10^5^ colony forming units (CFU) [13]. Detection of infections in the lung requires even larger numbers of bacteria, but infections such as these are not physiologically normal. Thus, significant enhancement of detection sensitivity could greatly improve the ability to study the progression of respiratory infection from the onset of infection under more relevant conditions.

Excitation intensities fall off exponentially as light propagates through homogeneous tissue; this loss typically plays a dominant role in detection sensitivity in whole-animal fluorescence imaging. Delivering the excitation light directly to the target area inside of an animal that is placed in a whole-animal imaging system could greatly enhance the ability to measure weak fluorescence signal from deep within the animal.

Fiber-optic based imaging has also become increasingly versatile in recent years as fiber components have decreased in size and gained functionality [14]. Flexible fiber-optic endoscopes, ranging in size of a millimeter up to a centimeter in diameter, have been used to image hollow cavity tissues such as the cervix or digestive tract [15–28]. A fiber-based microendoscope coupled into a whole-animal imaging system allows for excitation light to be delivered inside an animal model. This greatly enhances the intensity inside the animal, as the optical path length between the source and expected location of the fluorophore is minimized. In addition, such a system allows for multi-scale imaging, where microscopic imaging is achieved simultaneously with macroscopic whole-animal images. Although the use of fiber optic technology can be applied to a variety of biomedical applications where tissue is accessible by a thin optical fiber, the target application for this study is tuberculosis (TB), a major public health problem worldwide with an especially high prevalence in the developing world, where it is the leading cause of death.

TB, caused by bacillus *Mycobacterium tuberculosis* (Mtb), remains a major global health problem, infecting one-third of the world’s population [29]. The slow growth rate of Mtb limits progress toward understanding tuberculosis including diagnosis of infections and evaluation of therapeutic efficacy. Therefore, there is a growing need for more accurate and rapid diagnostic techniques in order to improve the sensitivity and specificity of the traditional methods for TB detection. A noninvasive biophotonic imaging technique to monitor bacterial dynamics in the host animal over time with high sensitivity would accelerate pre-clinical evaluation of novel vaccines and therapeutic agents [30]. To significantly enhance detection sensitivity of fluorescent mycobacteria in the mouse lung, we present the integration of an optical fiber into a whole-animal imaging system for intravital excitation of a fluorescent target deep within an animal.

## Methods

### Bacterial Strains and Growth Conditions

*Mycobacterium bovis* BCG (Bacillus Calmette Guerin) is not virulent in humans and was used as a model for tuberculosis in this study, since it is closely related to virulent strains. BCG is an attenuated form of live bovine tuberculosis bacillus and routinely used as a vaccine for TB. However, BCG retains the ability to infect and persist within mammalian tissue, similar to pathogenic mycobacteria [31]. Two BCG strains, BCG17 (fluorescent protein tdTomato-expressing BCG strain) and BCG39 (BCG strain carrying the same plasmid without tdTomato), were used. Construction of these strains is detailed in [12, 13, 32]. BCG strains were grown in 7H9 broth (Difco, Detroit, MI) supplemented with 0.5% glycerol, 10% OADC (oleic acid dextrose complex without catalase), and 0.05% Tween 80 (M-OADC-TW broth), or on Middlebrook 7H9 (BD, Franklin Lakes, NJ) supplemented with 10% OADC and 15 g/L Bacto agar (M-OADC agar), or on 7H11 selective media (BD).

tdTomato was selected as the fluorescent protein for tuberculosis during infection in mice for reasons described in [33] including its superior quantum yield as compared to the enhanced form of green fluorescent protein (EGFP) [34] and its long emission tail, going out to 700 nm, which makes this protein more suitable for *in vivo* imaging [5]. Also, tdTomato has sufficient photostability, which makes it stable throughout imaging with minimal photobleaching [5, 35, 36]. In addition, previous work by Y. Kong et al. demonstrated superior detection limits with tdTomato labeled BCG as opposed to BCG expressing EGFP [13]. Fluorescent systems in microscopy, pathology, and protein analyses are widely used for bacteria and other infectious agents [37–42]. Intravital excitation for whole-animal imaging enhances the potential use of current fluorescent reporters widely used in pre-clinical imaging studies.

### Animal Infections

All animal experiments in this study were approved by Texas A&M University Institutional Animal Care and Use Committee under animal use protocol number 2015–0222. Five- to seven-week old female BALB/C mice were used in all experiments. Mice were allowed to acclimate to the Biosafety Level 2 (BSL2) facilities for a week and fed AIN-93G diet (Harlan Teklad, Indianapolis, IN; TD.94045), a purified chlorophyll-free diet to reduce background autofluorescence [43–46], with *ad libitum* access to tap water. Total 42 mice were randomly grouped with six mice in a group. Groups of mice were infected by intratracheal instillation of 10^1^−10^6^ CFU BCG17 (tdTomato-expressing BCG strain) or 10^5^ CFU BCG39 (same plasmid without tdTomato) as a vector control. The detailed procedure of infection was described previously [47]. After 24 hours of infection, whole-body images were acquired using epi-illumination and intravital microendoscope illumination. Mice were sacrificed by injection of 100 μL Fatal-Plus Solution (Vortech Pharmaceuticals Ltd, Dearborn, MI, NDC; 0298-9373-68), and lungs were removed for *ex vivo* imaging. The excised lung tissues were homogenized and 10-fold serial dilutions of lung homogenates were plated on 7H11 selective media to enumerate bacterial CFU in the lung.

### Fluorescence Microendoscope

**Fig 1:**
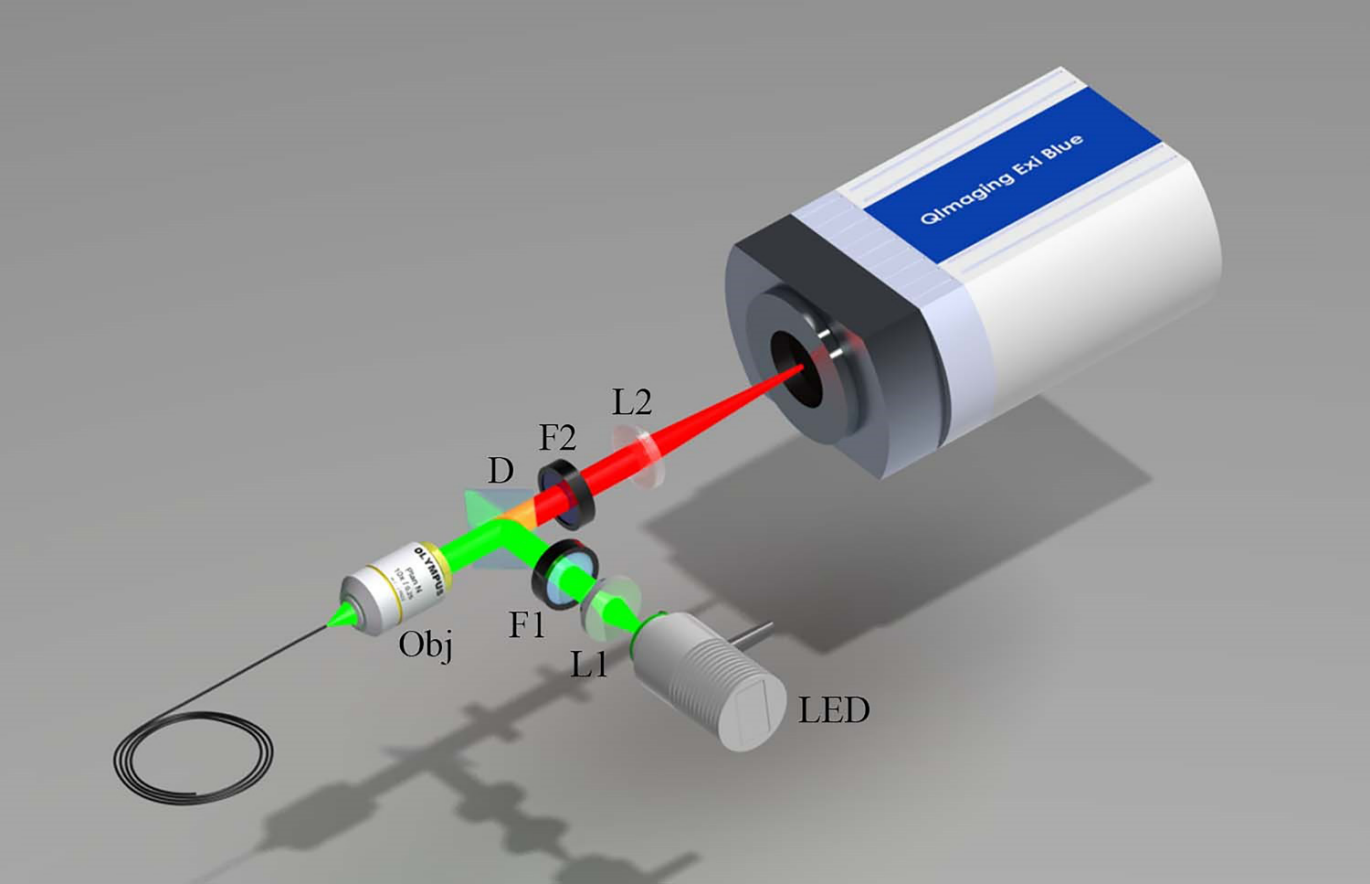
Illustration of the microendoscope system. LED: light emitting diode; L1: collimating lens; F1: excitation filter; D: dichroic beamsplitter; Obj: Microscope objective; F2: Emission filter; L2: focusing lens; FB: fiber bundle.

Fig 1 illustrates the fluorescence microendoscope that was constructed for bacterial detection. The basic design is similar to the system previously reported for the use in bacterial imaging *in situ* [33]. A light emitting diode (Thorlabs, Newton, NJ; M530L2) centered at 530 nm with a 31 nm bandwidth is used for fluorescence excitation. This wavelength was selected for excitation of tdTomato fluorescent protein (Ex: 554/Em: 581). Light from the diode is collimated, filtered with an excitation filter (Semrock, Rochester, NY; FF01-531/40) and dichroic mirror (Semrock; FF562-Di), and launched into a fiber bundle (Fujikura, Dudley, MA; FIGH-10-500N) by a 10x objective lens (Thorlabs; RMS10X). The 0.66 mm outer diameter fiber bundle consists of 10,000 individual optical fibers. A 3 μm core-to-core spacing limits the resolution, and the 450 μm active area determines the field of view of the microendoscope [48]. Excitation light is guided by the fiber bundle to its distal tip, which can then be inserted into an orifice of an animal model and placed in contact with internal organs. The typical output power from the fiber bundle was measured to be 300 μW. Fluorescence emission incident on the fiber bundle and within its angle of collection is relayed by the bundle, filtered by a longpass emission filter (Semrock; FF01-593/LP-25), and then imaged onto a scientific grade 1.45 megapixel CCD camera (QImaging, Surrey, BC, Canada; EXi Blue) with 6.45 μm x 6.45 μm pixel size.

### Integration of Microendoscope with Whole-Animal Imager

**Fig 2:**
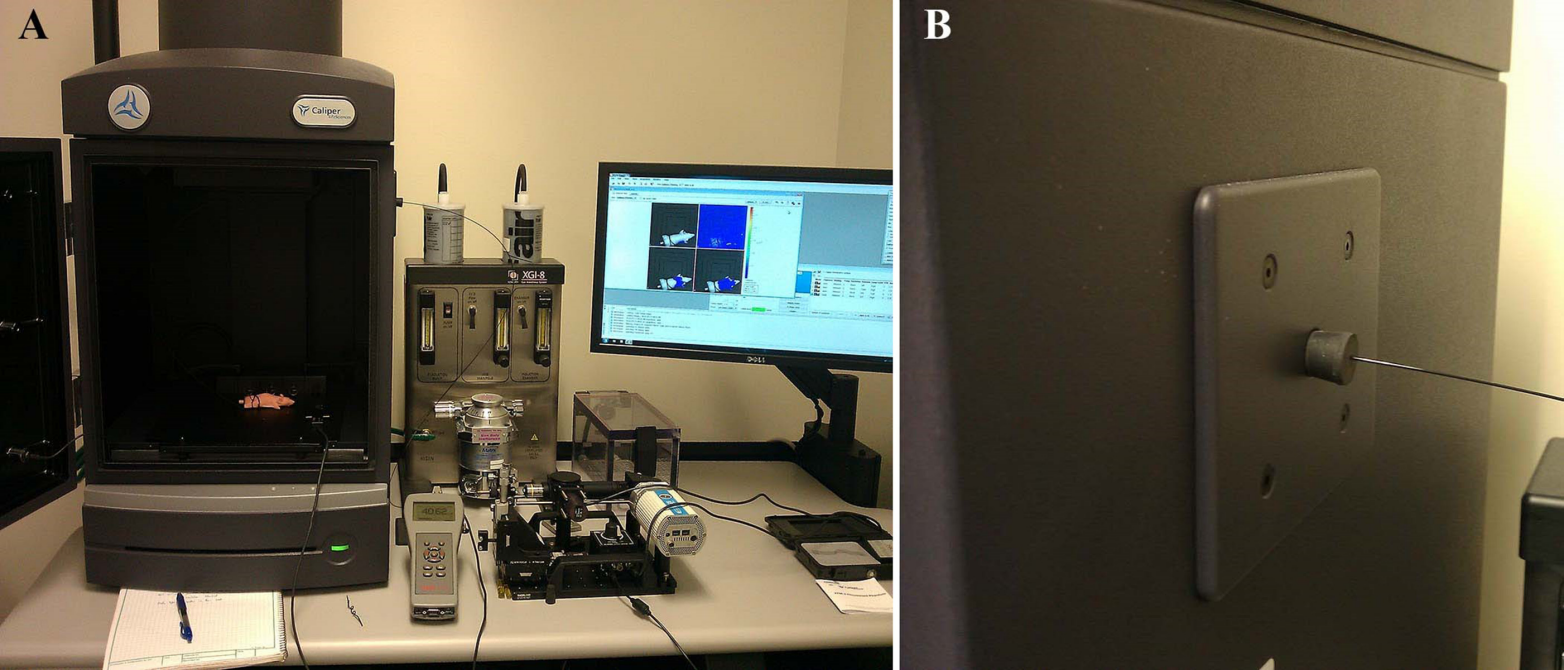
Integrated fluorescence imaging system. (A) Integration of the intravital fiber excitation source into the IVIS whole-animal imaging system. (B) The fiber is coupled via a test tube stopper placed into the access port of the IVIS system.

Multi-scale imaging of bacterial infections was achieved by integrating the microendoscope into a whole-animal optical imager (PerkinElmer, Waltham, MA; IVIS Lumina II). The fiber bundle was inserted into the whole-animal enclosure through an access port located on the side of the system, shown in Fig 2. To prevent any external light from entering the system, a 0.5 mm hole was drilled into a rubber stopper (VWR, Radnor, PA; 59580–069) which was then inserted into the access port opening. The fiber bundle was then inserted through the hole in the rubber stopper, as shown in Fig 2(B).

### *In Vivo* Animal Whole-Body Imaging

The detection of respiratory infections of BCG was used to evaluate the enhancement of fluorescence signal in whole-animal imaging using an intravital excitation source. All imaging was performed in a BSL2 laboratory in accordance with local safety regulations. Mouse fur was not removed for imaging; previous work demonstrated accurate localization and detection of tdTomato labeled BCG strains in mice that had fur [12, 13, 32]. A 22G x 1” intratracheal catheter (TERUMO Medical, Somerset, NJ; SURFLO® I.V. SR-OX2225CA) was inserted into the trachea of the mouse anesthetized with ketamine (100 mg/kg) (Henry Schein, Melville, NY) and xylazine (10 mg/kg) (Alfa Aesar, Haverhill, MA) mixture [49]. The mice were then placed on the IVIS stage in a ventral position, and the paws were loosely secured to the stage with tape. The lower abdomen was covered using a black cloth to block background fluorescence signal generated from gastrointestinal tract and kidney. The fiber bundle was inserted into the intratracheal catheter until it was in proximity with the tracheal wall and fluorescent signal could be observed using the microendoscope. Insertion of the fiber into the mouse airway was limited to the initial bifurcation of the lungs, as guiding the bundle beyond this point is difficult in the small space of a mouse lung. The fiber bundle was marked to ensure comparable insertion distance among animals and to help guide the fiber insertion in the vector control animals. The fiber bundle was also secured to the stage to ensure constant positioning of the fiber during imaging. The distal section of the fiber bundle, which comes in contact with the infected tissue, was thoroughly decontaminated with 70% isopropyl rubbing alcohol (Vi-Jon, Smyrna, TN) after imaging each animal to avoid cross-contamination.

Animal whole-body imaging was acquired by IVIS Lumina II using two different excitation sources–intravital and epi-illumination. First, the microendoscope was used for intravital excitation with the IVIS excitation filter wheel set to ‘block’ the IVIS illumination. Fluorescence images were collected with the IVIS emission filter set to 580, 600, 620, and 640 nm center wavelengths, as well as the “open” setting, which was used to visualize the illumination profile in the animal. The corresponding microscopic image of the lung tissue was also collected using the microendoscope CCD. Subsequently, the microendoscope source was switched off, and the IVIS excitation filter was set first to 535 nm for excitation of tdTomato fluorescence and then to 465 nm for autofluorescence. Epi-illumination images were captured using the same IVIS emission filter center wavelengths as for intravital excitation. Following whole-body imaging, the lungs were excised and imaged using epi-illumination with the same excitation and emission filter sets as whole-body imaging. For all images, IVIS acquisition settings were as follows: f/stop: 2, Binning: 8, Automatic exposure.

### Image Analysis

Manual spectral unmixing was performed using PerkinElmer Living Image software (version 4.3.1) in order to reduce the effects of tissue autofluorescence background and to quantify the tdTomato fluorescence signal detected by whole-body imaging with intravital or epi-illumination. For each animal, the spectrum for tdTomato fluorescence exiting the body from the lungs was estimated by subtracting the spectrum containing both tdTomato and tissue autofluorescence obtained from the mouse infected with tdTomato-expressing BCG strain from the tissue autofluorescence spectrum obtained from the vector control animal. Following spectral unmixing, fluorescence radiance was quantified over a pre-defined region of interest targeting the lungs.

### Statistical Analysis

Statistical analysis was performed using GraphPad Prism software (Version 6). A nonparametric statistical method, Kruskal-Wallis for multiple comparisons with the Bonferroni posttest (Dunn’s procedure), was used to determine statistical significance compared to the vector control [50–52]. P < 0.05 was considered significant. To test the correlation of CFU and fluorescence signals, a Pearson correlation test was performed. Linear regression analysis was also applied to determine the relationship between CFU and fluorescence signals.

## Results and Discussion

**Fig 3:**
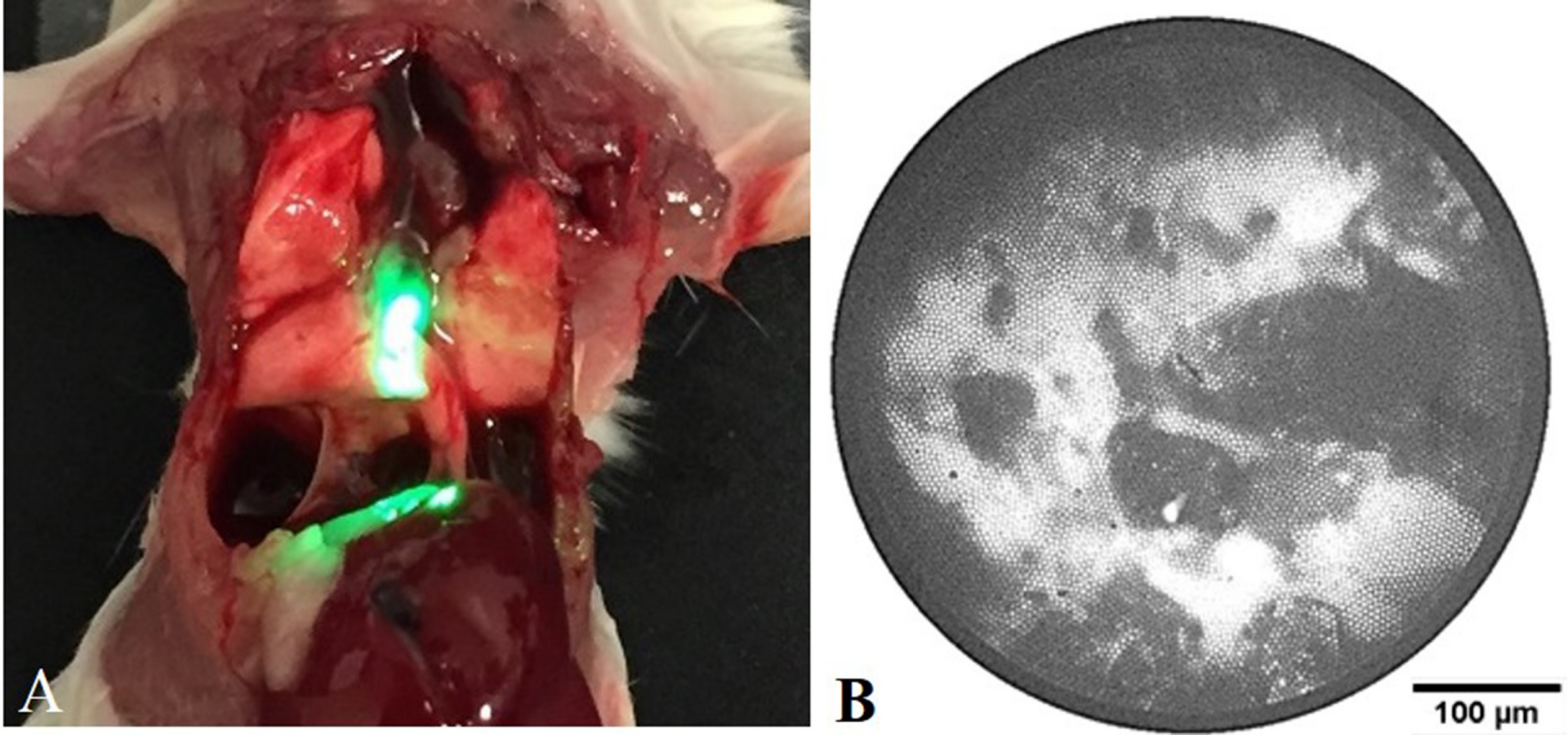
Intravital excitation illumination via microendoscope in the mouse airway. (A) Image of sacrificed mouse with open chest wall and intravital illumination showing the proximal placement of the fiber bundle in the trachea. (B) Example fiber bundle image of BCG17 bacterial fluorescence collected with the microendoscope CCD. The microendoscope image is used to guide placement of the intravital source in contact with tissue prior to IVIS imaging.

While providing the ability to acquire both macroscopic and microscopic images of bacterial infections from the same animal, the combination of the two optical systems alleviates some of the individual limitations of each. First, the IVIS CCD can be used to localize and track the fiber bundle position inside the animal, allowing for proper positioning of the distal tip prior to data collection. More sophisticated whole-animal imaging systems with optical tomography or x-ray imaging capabilities could allow more accurate determination of the fiber tip location. Additionally, the microendoscope source can serve as an intravital excitation source for the whole-animal fluorescence imager. By delivering the excitation light internally, the attenuation of the excitation light by tissue structures such as the chest wall is greatly decreased, allowing for an increase in the delivery of excitation light inside the region of interest. Prior to *in vivo* imaging using intravital excitation, we evaluated intravital excitation illumination in the lung of mouse. Fig 3(A) shows a mouse with its chest wall opened after sacrifice, exposing the lungs and demonstrating the origin of the intravital excitation light within the animal’s body. Fig 3(B) depicts a typical microendoscope image obtained from the fiber bundle. The fiber bundle pattern is superimposed on the fluorescence signal acquired from the tip of the microendoscope.

### *In Vivo* Whole-Body Imaging Using Intravital Fiber Excitation

**Fig 4:**
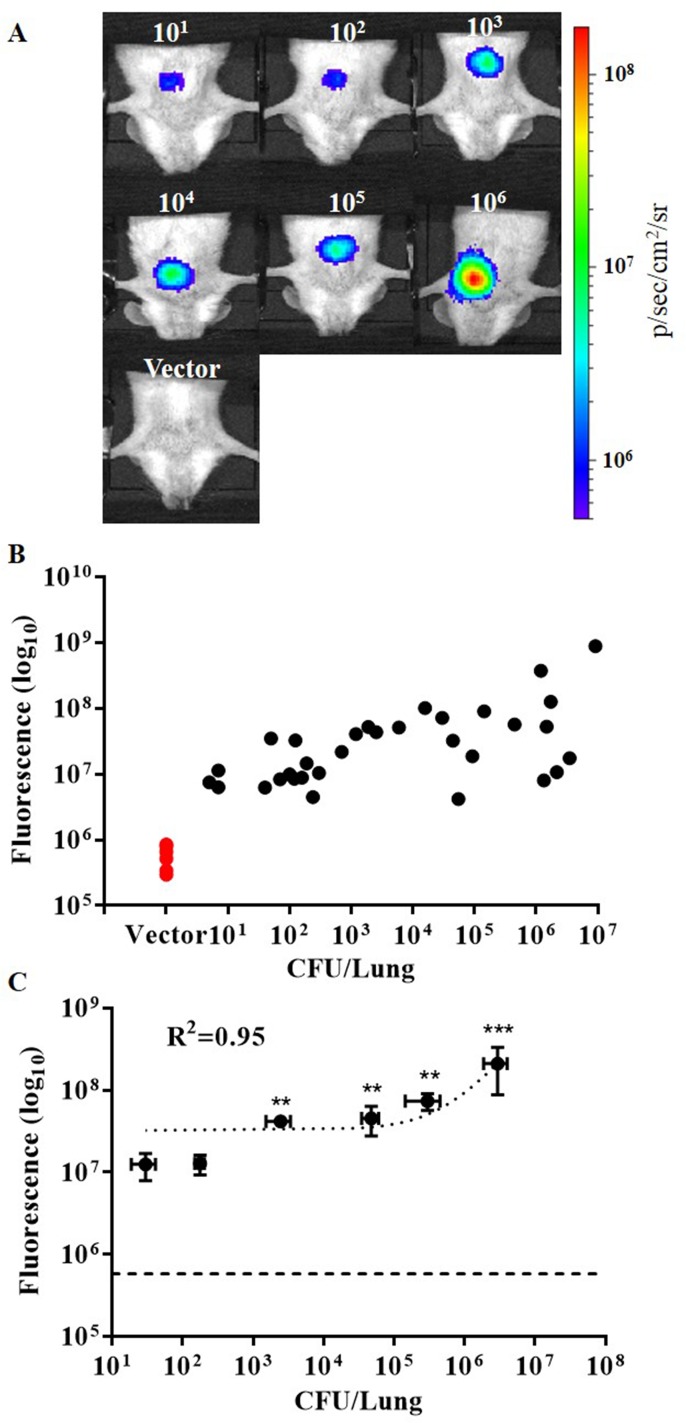
Intravital fluorescence excitation of tdTomato expressing BCG after intratracheal pulmonary infection of mice. (A) Representative whole-body images acquired using integrated microendoscope (intravital fiber excitation) at 24 hr post-infection with 10^1^−10^6^ colony forming units (CFU) BCG17 (tdTomato expressing BCG strain) and 10^5^ CFU BCG39 (BCG containing the same vector that does not express tdTomato (Vector)). (B) Scatter plot of fluorescence signal for each animal imaged as compared to actual CFU in lung homogenates from the same mouse. (C) Correlation of fluorescent signal to number of bacterial CFU in lung homogenates. Error bars represent the standard error for each sample group. **p‐value < 0.01, ***p‐value < 0.001: significantly different from fluorescence of vector control group (horizontal dashed line in C) calculated by non-parametric Kruskal-Wallis test with the Bonferroni posttest. All images and measurements represent tdTomato contribution to signal after spectral unmixing.

Representative whole-body images acquired using the intravital fiber excitation are shown in Fig 4(A) for mice infected with 10^1^ to 10^6^ CFU BCG17, as well as a representative image of a 10^5^ CFU infection of BCG vector control. Images with signal levels close to the mean value for the group were considered representative. All images were acquired using identical imaging parameters. Fluorescence images have been scaled such that no signal was present in the vector control for better comparison. Fluorescence signal was quantified by measuring fluorescence intensity over the chest region of interest. Detected fluorescence signal increased with bacterial CFU and minimal signal was observed in the vector control group (Fig 4). We found that intravital excitation could be used to detect pulmonary infection levels down to 10^3^ CFU with a significant difference from the vector control (P < 0.01). A linear regression fit was applied to assess correlation of the average fluorescence for each group with average CFU in lung homogenates. Fluorescence signal correlates well with bacterial CFU, with R^2^ = 0.945 (P = 0.0011), demonstrating the ability to quantify bacterial CFU using intravital excitation.

**Fig 5:**
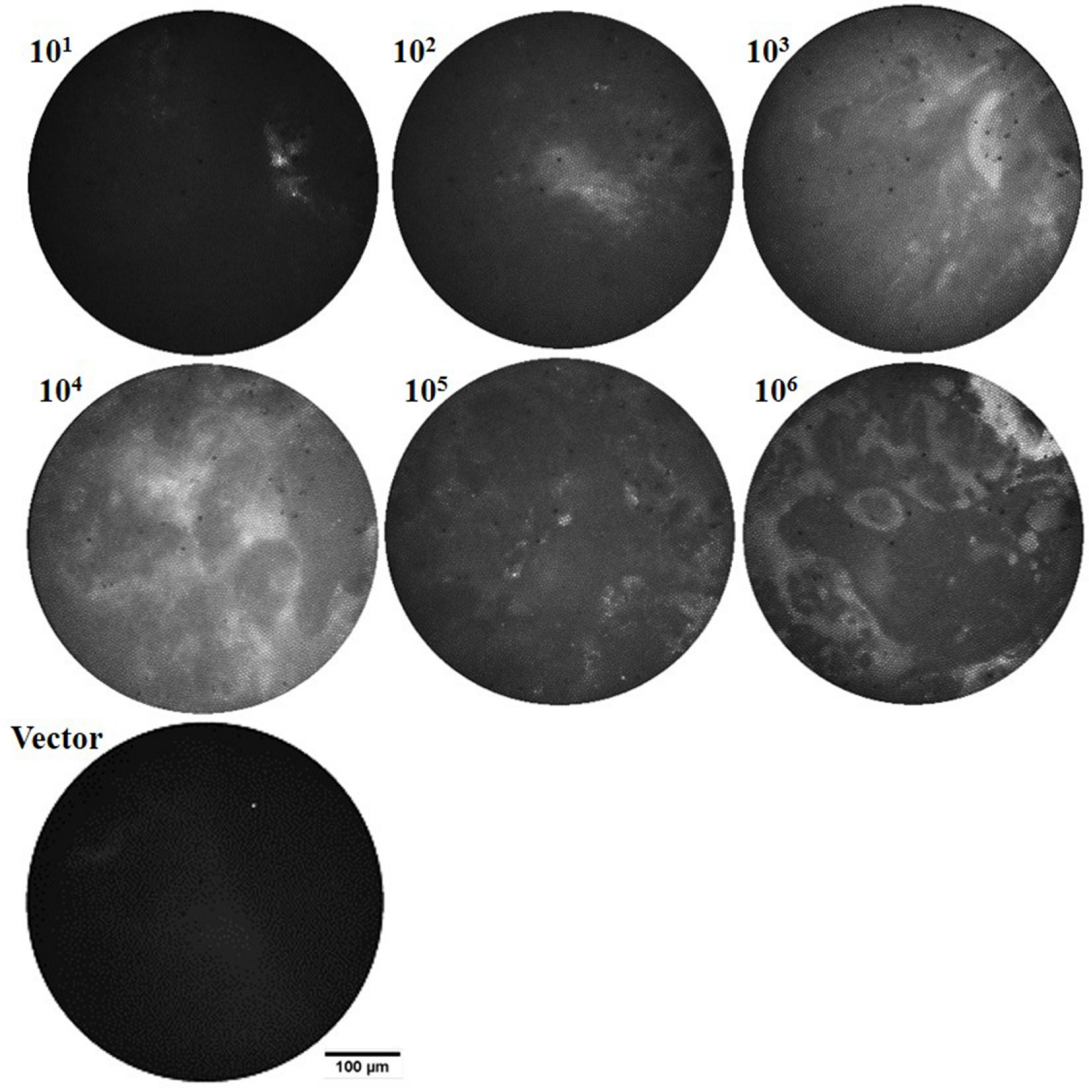
*In vivo* microendoscope images provide feedback for positioning the intravital excitation source and can allow a qualitative evaluation of bacterial loads in the lungs. Representative *in vivo* fiber microendoscopy images of infected mice lungs, 24 hr post-infection with 10^1^−10^6^ colony forming units (CFU) BCG17 (tdTomato expressing BCG strain) and 10^5^ CFU BCG39 (BCG containing the same vector that does not express tdTomato (Vector)).

Representative microscopic images acquired using the microendoscope CCD camera are shown in Fig 5 for the mice infected with different concentrations of BCG17 (10^1^−10^6^ CFU), as well as a representative image of the 10^5^ CFU vector control. Fluorescence signal captured by the microendoscope increased with bacterial numbers, particularly for mice with >10^4^ CFU in lung homogenates. However, microendoscopic fluorescence is dependent on positioning of the intratracheal catheter and microendoscope fiber tip within the airway. Microendoscope images are primarily used to ensure fiber tip contact with tissue, which results in enhanced intravital excitation for whole-animal imaging. Bacteria are unlikely to be located precisely at the surface of the microendoscope following 24 hr incubation; therefore, spatial variation within a single microendoscope image may be attributed to scattering of fluorescence emission by tissue and mucous structure at the surface of the microendoscope tip.

### *In Vivo* Whole-Body Imaging Using IVIS Epi-Illumination

**Fig 6:**
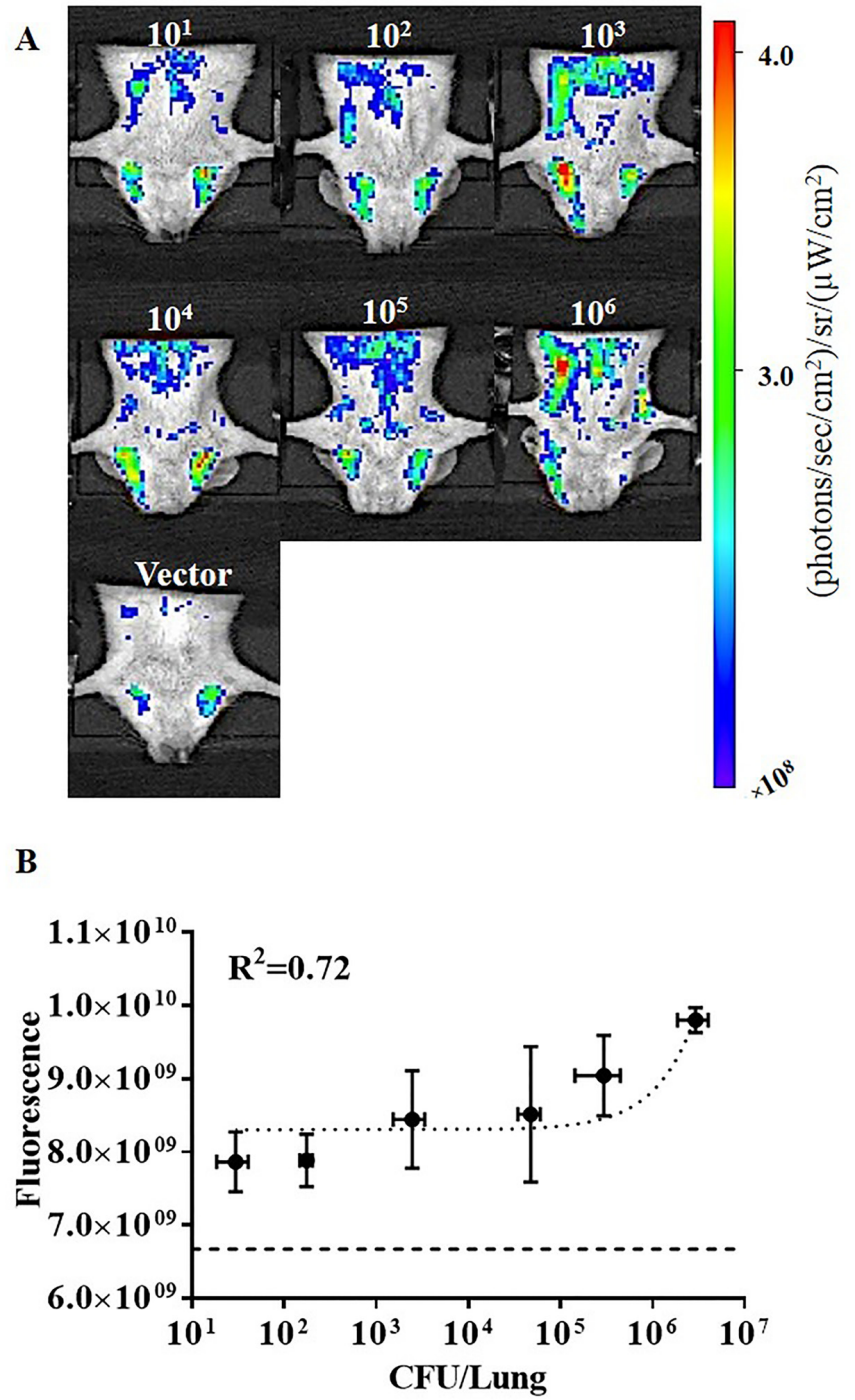
*In vivo* whole-body imaging of infected mice using IVIS epi-illumination. (A) Representative images of bacterial infection at 24 hr post-infection with 10^1^ to 10^6^ colony forming units (CFU) BCG17 (tdTomato expressing BCG strain) and 10^5^ CFU BCG39 (BCG containing the same vector that does not express tdTomato (Vector)). (B) Correlation of fluorescence signal in mouse whole-body images versus CFU in lung homogenates from the same animal. Error bars represent the standard error for each sample group. Horizontal dashed line in (B) represents the average signal for the vector control group. All images and measurements represent tdTomato contribution to signal after spectral unmixing.

To provide a comparison between whole animal imaging using intravital excitation and standard epi-illumination, images were acquired using the IVIS epi-illumination excitation and detection. Representative images for infections of 10^1^ to 10^6^ CFU BCG17 are shown in Fig 6(A). A representative image of the 10^5^ CFU vector control is shown in Fig 6(A). Images with signal levels close to the mean value for the group were considered representative. All images were acquired using identical imaging parameters. Epi-illumination in whole-animal imaging is unable to differentiate between vector control group and infected groups with any statistical significance, even for the highest inoculation dose (P = 0.054). Although the fluorescence intensity for BCG17 infected groups is not significantly higher than the vector group, even after spectral unmixing, the detected signal correlates well with CFU numbers. Comparing signal to background levels for epi-illumination (Fig 6(B)) and intravital illumination (Fig 4(C)), shows that signal to background level for epi-illumination is less than 2× for all infections; whereas, signal to background level in intravital excitation ranges from 10× to more than 100× with increasing bacterial load.

### *Ex Vivo* Lung Tissue Imaging

**Fig 7:**
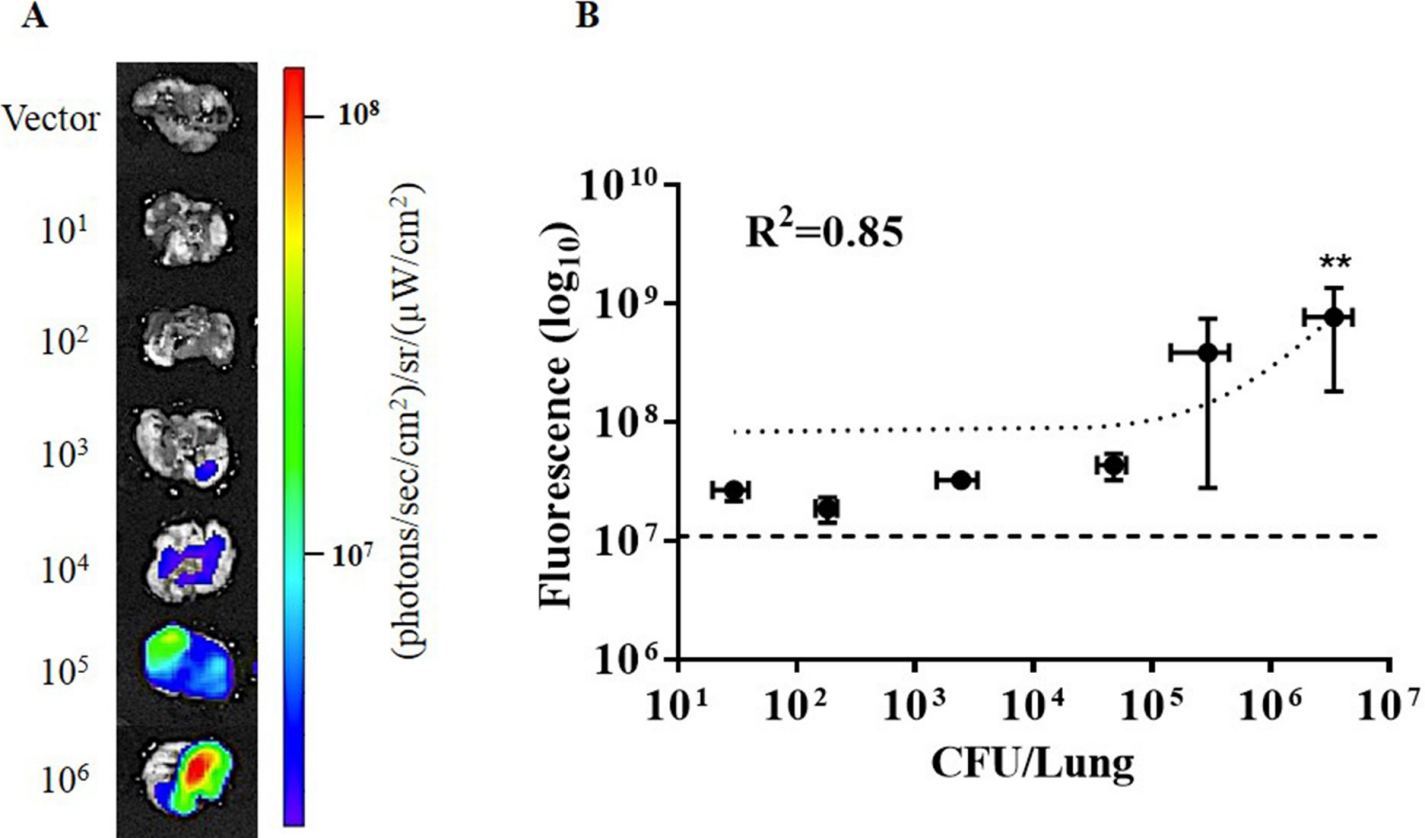
Epi-illumination images of excised mouse lungs infected with tdTomato-expressing BCG or BCG with vector backbone. (A) Representative images of excised lungs infected with 10^1^ to 10^6^ colony forming units (CFU) BCG17 (tdTomato expressing BCG strain) and 10^5^ CFU BCG39 (BCG containing the same vector that does not express tdTomato (Vector)). (B) Correlation of fluorescence signal in *ex vivo* images of lungs and CFU in lung homogenates from the same animal. Error bars represent the standard error for each sample group. ** p‐value < 0.01: significantly different from fluorescence of vector control group (horizontal dashed line in B) calculated by non-parametric Kruskal-Wallis test with the Bonferroni posttest. All images and measurements represent tdTomato contribution to signal after spectral unmixing.

To avoid skin autofluorescence and improve penetration of epi-illumination excitation light to the bacteria within the lung, infected lungs were excised and imaged using IVIS epi-illumination. The same acquisition filter settings and manual spectral unmixing method were used as whole-body imaging using IVIS epi-illumination. As shown in Fig 7, fluorescence signal correlates with increased bacterial numbers as quantified by CFU in lung homogenates (R^2^ = 0.851, P = 0.0088). However, detection of infection was significant only in the group infected with 10^6^ CFU BCG17 as compared to the vector control group (P = 0.0030). Epi-illumination imaging of lungs *ex vivo* enhances signal to background compared to *in vivo* by the same technique. Internal excitation in excised lungs is not straightforward due to the complexity and small size of the trachea and the potential for collapse of the lungs upon penetration of pleura; however, intravital excitation combined with whole-animal imaging was found to be more sensitive than *ex vivo* epi-illumination.

## Conclusions

We have developed a novel system to sensitively detect bacterial infection in living animals. By integrating a fiber-based microendoscope into the IVIS whole-animal imaging system, we enabled intravital excitation of fluorescent bacteria within the mouse lung. Using intravital excitation and whole-animal fluorescence imaging, we decreased the level of detection of tdTomato-expressing bacteria in the lungs of living mice to 10^3^ CFU; whereas, epi-illumination for whole-animal imaging was unable to detect infections with as many as 10^6^ CFU.

Intravital excitation circumvents the expected absorption of light by the tissue present in the chest wall of mammals. Furthermore, autofluorescence is significantly reduced by illuminating from within the body, similar to the autofluorescence reduction observed with transillumination using whole-body imaging systems. Transillumination, where the light source and detector are located on opposite sides of the animal, provides enhanced detection sensitivity as compared to epi-illumination, most likely due to reflected autofluorescence. As excitation and emission light must be transmitted through the animal to the fluorescent source being detected, these systems inherently are impacted by characteristics of the tissue deep inside the animal and are less affected by the depth of the fluorophore. Therefore, transillumination is more ideally suited for detection of fluorescence from the respiratory system. Intravital excitation has the potential to be more sensitive yet, due to the more direct delivery of excitation light, which is shorter in wavelength than emission light and more dramatically impacted by depth.

While the fiber microendoscope used for these experiments employed a fiber bundle to deliver excitation light to the lungs, a simple large core, multimode fiber could be used in its place. This would allow for additional increases in excitation power delivery. In addition, the system could be further miniaturized and designed to operate solely on battery power. This would allow for imaging inside whole-animal imaging systems that might lack an access port, though this was not necessary for our current system, since the IVIS Lumina II has an access port.

Insertion of the intratracheal catheter and microendoscope into the airway must be performed with great care and caution to avoid tissue damage or animal suffering. There was no evidence of tissue damage due to optical fiber placement. The insertion of an object into the airway has the potential to disturb or artificially spread the infection within the airway. To avoid contamination between animals, the tip of the optical fiber is carefully decontaminated with isopropyl alcohol, after imaging each animal.

A greater understanding of the illumination profile generated within the body by the internal excitation is needed to better understand the physical limitations of intravital excitation. In this study, the fiber bundle excitation source is placed at the tracheal bifurcation and directed towards the base of the lungs. After 24 hours post-infection bacteria may not be present in the trachea or bronchi. Using intravital excitation in mice and other small animals, the highly scattering nature of the lung tissue is exploited to illuminate the entire chest cavity. Excitation light from the intravital source is visible through the chest wall.

Imaging data presented here was acquired 24 hours after bacterial infection. Intravital excitation should be assessed to monitor dynamics of bacterial load over longer time periods. Typically, a granuloma forms at approximately five weeks or later post-infection. The impact of the immune response or granuloma formation on intravital excitation of bacteria within a granuloma would be an interesting avenue of further study, as well as different aspects of pathology that might arise during later stages of disease [53–56].

Beyond the application of this technology to detection of mycobacteria in the lungs, intravital excitation may be applied to enhance sensitivity of fluorescent marker detection in other deep tissues, such as within the gastrointestinal tract. The fiber bundle microendoscope or optical fiber excitation source can be passed through other natural orifices or inserted into the body through a needle in a minimally invasive procedure. We envision a number of additional applications for this technology that could allow improvements in imaging sensitivity of not only infectious diseases, but also other diseases of significant importance that can be evaluated with optical imaging.

## References

[1] Yuste R. Fluorescence microscopy today. Nature Methods. 2005;2(12):902–4. 16299474 10.1038/nmeth1205-902

[2] Baker M. Whole-animal imaging: The whole picture. Nature. 2010;463(7283):977–80. 10.1038/463977a 20164931

[3] Leblond F, Davis SC, Valdés PA, Pogue BW. Pre-clinical whole-body fluorescence imaging: Review of instruments, methods and applications. Journal of Photochemistry and Photobiology B: Biology. 2010;98(1):77–94. 10.1016/j.jphotobiol.2009.11.007 PMC3678966 20031443

[4] Ntziachristos V. Fluorescence molecular imaging. Annual Review of Biomedical Engineering. 2006;8:1–33. 16834550 10.1146/annurev.bioeng.8.061505.095831

[5] Shaner NC, Steinbach PA, Tsien RY. A guide to choosing fluorescent proteins. Nature Methods. 2005;2(12):905–9. . 16299475 10.1038/nmeth819

[6] Billinton N, Knight AW. Seeing the wood through the trees: a review of techniques for distinguishing green fluorescent protein from endogenous autofluorescence. Analytical Biochemistry. 2001;291(2):175–97. 11401292 10.1006/abio.2000.5006

[7] Wagnieres GA, Star WM, Wilson BC. In vivo fluorescence spectroscopy and imaging for oncological applications. Photochemistry and Photobiology. 1998;68(5):603–32. 9825692

[8] Ntziachristos V, Tung C-H, Bremer C, Weissleder R. Fluorescence molecular tomography resolves protease activity in vivo. Nature Medicine. 2002;8(7):757–61. 12091907 10.1038/nm729

[9] Tamgüney G, Francis KP, Giles K, Lemus A, DeArmond SJ, Prusiner SB. Measuring prions by bioluminescence imaging. Proceedings of the National Academy of Sciences. 2009;106(35):15002–6. 10.1073/pnas.0907339106 PMC2736416 19706444

[10] Chen J, Tung C-H, Mahmood U, Ntziachristos V, Gyurko R, Fishman MC, et al In vivo imaging of proteolytic activity in atherosclerosis. Circulation. 2002;105(23):2766–71. 12057992 10.1161/01.cir.0000017860.20619.23

[11] Sosnovik DE, Nahrendorf M, Deliolanis N, Novikov M, Aikawa E, Josephson L, et al Fluorescence tomography and magnetic resonance imaging of myocardial macrophage infiltration in infarcted myocardium in vivo. Circulation. 2007;115(11):1384–91. 17339546 10.1161/CIRCULATIONAHA.106.663351

[12] Kong Y, Yao H, Ren H, Subbian S, Cirillo SL, Sacchettini JC, et al Imaging tuberculosis with endogenous β-lactamase reporter enzyme fluorescence in live mice. Proceedings of the National Academy of Sciences. 2010;107(27):12239–44. 10.1073/pnas.1000643107 PMC2901431 20566877

[13] Kong Y, Subbian S, Cirillo SLG, Cirillo JD. Application of optical imaging to study of extrapulmonary spread by tuberculosis. Tuberculosis. 2009;89, Supplement 1(0):S15–S7. 10.1016/S1472-9792(09)70006-X 20006298 PMC4137470

[14] Flusberg BA, Cocker ED, Piyawattanametha W, Jung JC, Cheung EL, Schnitzer MJ. Fiber-optic fluorescence imaging. Nature Methods. 2005;2(12):941–50. 16299479 10.1038/nmeth820 PMC2849801

[15] Oh G, Chung E, Yun SH. Optical fibers for high-resolution in vivo microendoscopic fluorescence imaging. Optical Fiber Technology. 2013;19(6):760–71.

[16] Miller SJ, Lee CM, Joshi BP, Gaustad A, Seibel EJ, Wang TD. Targeted detection of murine colonic dysplasia in vivo with flexible multispectral scanning fiber endoscopy. Journal of Biomedical Optics. 2012;17(2):0211031–02110311. 10.1117/1.JBO.17.2.021103 PMC3380821 22463021

[17] Choi Y, Yoon C, Kim M, Yang TD, Fang-Yen C, Dasari RR, et al Scanner-free and wide-field endoscopic imaging by using a single multimode optical fiber. Physical Review Letters. 2012;109(20):203901 23215488 10.1103/PhysRevLett.109.203901 PMC4001713

[18] Lee CM, Engelbrecht CJ, Soper TD, Helmchen F, Seibel EJ. Scanning fiber endoscopy with highly flexible, 1 mm catheterscopes for wide‐field, full‐color imaging. Journal of Biophotonics. 2010;3(5‐6):385–407. 10.1002/jbio.200900087 20336702 PMC3163080

[19] Zhang Y, Akins ML, Murari K, Xi J, Li M-J, Luby-Phelps K, et al A compact fiber-optic SHG scanning endomicroscope and its application to visualize cervical remodeling during pregnancy. Proceedings of the National Academy of Sciences. 2012;109(32):12878–83. 10.1073/pnas.1121495109 PMC3420182 22826263

[20] Bao H, Boussioutas A, Jeremy R, Russell S, Gu M. Second harmonic generation imaging via nonlinear endomicroscopy. Optics Express. 2010;18(2):1255–60. 10.1364/OE.18.001255 20173949

[21] Brown CM, Rivera DR, Pavlova I, Ouzounov DG, Williams WO, Mohanan S, et al In vivo imaging of unstained tissues using a compact and flexible multiphoton microendoscope. Journal of Biomedical Optics. 2012;17(4):0405051–3. 10.1117/1.JBO.17.4.040505 PMC3382343 22559671

[22] Muldoon TJ, Anandasabapathy S, Maru D, Richards-Kortum R. High-resolution imaging in Barrett's esophagus: a novel, low-cost endoscopic microscope. Gastrointestinal Endoscopy. 2008;68(4):737–44. 10.1016/j.gie.2008.05.018 18926182 PMC2869299

[23] Keahey P, Tkaczyk T, Schmeler K, Richards-Kortum R. Optimizing modulation frequency for structured illumination in a fiber-optic microendoscope to image nuclear morphometry in columnar epithelium. Biomedical Optics Express. 2015;6(3):870–80. 10.1364/BOE.6.000870 25798311 PMC4361441

[24] Iftimia N, Iyer AK, Hammer DX, Lue N, Mujat M, Pitman M, et al Fluorescence-guided optical coherence tomography imaging for colon cancer screening: a preliminary mouse study. Biomedical Optics Express. 2012;3(1):178–91. 10.1364/BOE.3.000178 22254178 PMC3255336

[25] Jabbour JM, Saldua MA, Bixler JN, Maitland KC. Confocal endomicroscopy: instrumentation and medical applications. Annals of Biomedical Engineering. 2012;40(2):378–97. 10.1007/s10439-011-0426-y 21994069 PMC3710661

[26] Lorenser D, Quirk BC, Auger M, Madore W-J, Kirk RW, Godbout N, et al Dual-modality needle probe for combined fluorescence imaging and three-dimensional optical coherence tomography. Optics Letters. 2013;38(3):266–8. 10.1364/OL.38.000266 23381406

[27] Cheng S, Rico-Jimenez JJ, Jabbour J, Malik B, Maitland KC, Wright J, et al Flexible endoscope for continuous in vivo multispectral fluorescence lifetime imaging. Optics Letters. 2013;38(9):1515–7. 10.1364/OL.38.001515 23632536 PMC3702045

[28] Pierce M, Yu D, Richards-Kortum R. High-resolution Fiber-optic Microendoscopy for in situ Cellular Imaging. Journal of Visualized Experiments. 2011;(47):e2306 10.3791/2306 PMC3182629 21248707

[29] Global Tuberculosis Report. World Health Organization, 2014.

[30] Andreu N, Zelmer A, Wiles S. Noninvasive biophotonic imaging for studies of infectious disease. FEMS Microbiology Reviews. 2011;35(2):360–94. 10.1111/j.1574-6976.2010.00252.x 20955395 PMC3084502

[31] Gordon S, Keshav S, Stein M. BCG-induced granuloma formation in murine tissues. Immunobiology. 1994;191(4):369–77. 7713550 10.1016/S0171-2985(11)80442-0

[32] Kong Y, Akin AR, Francis KP, Zhang N, Troy TL, Xie H, et al Whole‐Body Imaging of Infection Using Fluorescence. Current Protocols in Microbiology. 2011:2C. 3.1–2C. 3.21. 10.1002/9780471729259.mc02c03s21 21538304

[33] Mufti N, Kong Y, Cirillo JD, Maitland KC. Fiber optic microendoscopy for preclinical study of bacterial infection dynamics. Biomedical Optics Express. 2011;2(5):1121–34. 10.1364/BOE.2.001121 21559125 PMC3087570

[34] Goldgeier M, Fox CA, Zavislan JM, Harris D, Gonzalez S. Noninvasive imaging, treatment, and microscopic confirmation of clearance of basal cell carcinoma. Dermatologic Surgery. 2003;29(3):205–10. 12614409 10.1046/j.1524-4725.2003.29050.x

[35] Shaner NC, Lin MZ, McKeown MR, Steinbach PA, Hazelwood KL, Davidson MW, et al Improving the photostability of bright monomeric orange and red fluorescent proteins. Nature Methods. 2008;5(6):545–51. 10.1038/nmeth.1209 18454154 PMC2853173

[36] Gee JM, Smith NA, Fernandez FR, Economo MN, Brunert D, Rothermel M, et al Imaging activity in neurons and glia with a Polr2a-based and cre-dependent GCaMP5G-IRES-tdTomato reporter mouse. Neuron. 2014;83(5):1058–72. 10.1016/j.neuron.2014.07.024 25155958 PMC4156920

[37] Dedecker P, Mo GC, Dertinger T, Zhang J. Widely accessible method for superresolution fluorescence imaging of living systems. Proceedings of the National Academy of Sciences. 2012;109(27):10909–14. 10.1073/pnas.1204917109 PMC3390831 22711840

[38] Chudakov DM, Matz MV, Lukyanov S, Lukyanov KA. Fluorescent proteins and their applications in imaging living cells and tissues. Physiological Reviews. 2010;90(3):1103–63. 10.1152/physrev.00038.2009 20664080

[39] Capoulade J, Wachsmuth M, Hufnagel L, Knop M. Quantitative fluorescence imaging of protein diffusion and interaction in living cells. Nature Biotechnology. 2011;29(9):835–9. 10.1038/nbt.1928 21822256

[40] Sevick-Muraca E. Translation of near-infrared fluorescence imaging technologies: emerging clinical applications. Annual Review of Medicine. 2012;63:217–31. 10.1146/annurev-med-070910-083323 22034868

[41] Bird-Lieberman EL, Neves AA, Lao-Sirieix P, O'Donovan M, Novelli M, Lovat LB, et al Molecular imaging using fluorescent lectins permits rapid endoscopic identification of dysplasia in Barrett's esophagus. Nature Medicine. 2012;18(2):315–21. 10.1038/nm.2616 22245781

[42] White AG, Fu N, Leevy WM, Lee J-J, Blasco MA, Smith BD. Optical imaging of bacterial infection in living mice using deep-red fluorescent squaraine rotaxane probes. Bioconjugate Chemistry. 2010;21(7):1297–304. 10.1021/bc1000998 20536173 PMC2912452

[43] Troy T, Jekic-McMullen D, Sambucetti L, Rice B. Quantitative comparison of the sensitivity of detection of fluorescent and bioluminescent reporters in animal models. Molecular Imaging. 2004;(3):9–23. 15142408 10.1162/15353500200403196

[44] MacLaurin SA, Bouchard M, Dwyer P, Levenson R, Mansfield J, Krucker T, editors. Reduction of skin and food autofluorescence in different mouse strains through diet changes. Poster, Society for Molecular Imaging, Annual Meeting, Hawaii; 2006.

[45] Bhaumik S, DePuy J, Klimash J. Strategies to minimize background autofluorescence in live mice during noninvasive fluorescence optical imaging. Lab Animal. 2007;36(8):40–3. 17721532 10.1038/laban0907-40

[46] Inoue Y, Izawa K, Kiryu S, Tojo A, Ohtomo K. Diet and abdominal autofluorescence detected by in vivo fluorescence imaging of living mice. Molecular Imaging. 2008;7(1):21–7. 18384720

[47] Kong Y, Akin AR, Francis KP, Zhang N, Troy TL, Xie H, et al Whole-Body Imaging of Infection Using Fluorescence Current Protocols in Microbiology: John Wiley & Sons, Inc.; 2011. 10.1002/9780471729259.mc02c03s21 21538304

[48] Muldoon TJ, Pierce MC, Nida DL, Williams MD, Gillenwater A, Richards-Kortum R. Subcellular-resolution molecular imaging within living tissue by fiber microendoscopy. Optics Express. 2007;15(25):16413–23. 19550931 10.1364/oe.15.016413 PMC3065245

[49] Chang MH, Cirillo SLG, Cirillo JD. Using Luciferase to Image Bacterial Infections in Mice. Journal of Visualized Experiments. 2011;(48):e2547 10.3791/2547 PMC3197412 21372790

[50] Dunnett CW. A multiple comparison procedure for comparing several treatments with a control. Journal of the American Statistical Association. 1955;50(272):1096–121.

[51] Demšar J. Statistical comparisons of classifiers over multiple data sets. The Journal of Machine Learning Research. 2006;7:1–30.

[52] García S, Fernández A, Luengo J, Herrera F. Advanced nonparametric tests for multiple comparisons in the design of experiments in computational intelligence and data mining: Experimental analysis of power. Information Sciences. 2010;180(10):2044–64.

[53] Travis LW, Hybels RL, Newman M. Tuberculosis of the larynx. The Laryngoscope. 1976;86(4):549–58. 1263725 10.1288/00005537-197604000-00011

[54] Gupta P, Kolluri SV, Chandramouli B, Venkataramana N, Das B. Calvarial tuberculosis: a report of two cases. Neurosurgery. 1989;25(5):830–3. 2586738

[55] Bernaerts A, Vanhoenacker F, Parizel P, Van Goethem J, Van Altena R, Laridon A, et al Tuberculosis of the central nervous system: overview of neuroradiological findings. European Radiology. 2003;13(8):1876–90. 12942288 10.1007/s00330-002-1608-7

[56] Torres RM, Calonge M. Macular edema as the only ocular finding of tuberculosis. American Journal of Ophthalmology. 2004;138(6):1048–9. 15629302 10.1016/j.ajo.2004.06.020

